# Beyond Diagnosis: The Multifaceted Relationship Between Gender Dysphoria and Pervasive Developmental Disorders

**DOI:** 10.1192/j.eurpsy.2025.1162

**Published:** 2025-08-26

**Authors:** M.-M. Mihai, M. Moise, T. Costea

**Affiliations:** 1 Darent Valley Hospital, Dartford and Gravesham NHS Trust, Dartford, United Kingdom; 2 University of Medicine and Pharmacy Carol Davila; 3 Professor Doctor Alexandru Obregia Clinical Hospital of Psychiatry, Bucharest, Romania

## Abstract

**Introduction:**

This case series examines five adolescents with both gender dysphoria and pervasive developmental conditions, highlighting the social, familial, and psychological challenges involved. The cases reveal how these conditions intersect, shaping identity, social interactions, and family dynamics. Findings suggest a trend toward isolation and virtual spaces for acceptance, with limited family support often exacerbating isolation. Integrated therapeutic approaches addressing both gender dysphoria and developmental conditions are recommended to improve mental health and self-acceptance.

**Objectives:**

To explore the diversity of gender dysphoria manifestations within pervasive developmental disorders.

**Methods:**

We analysed five cases of adolescents from Professor Doctor Alexandru Obregia Clinical Hospital of Psychiatry in Bucharest, each diagnosed with both gender dysphoria and a pervasive developmental disorder. Each case highlights unique psychological and social factors influencing the adolescents’ identities and interactions. Patients were monitored over an average period of one year to observe developments and responses to therapeutic interventions.

**Results:**

The cases illustrate diverse expressions of gender dysphoria among adolescents with pervasive developmental disorders:

Case 1: 12-year-old with Asperger’s syndrome and gender dysphoria, with interests and social withdrawal shaped by online interactions, further isolating her.

Case 2: 15-year-old with severe depression and Asperger’s syndrome, marked by social withdrawal, a strong attachment to solitary pursuits, and an identity struggle.

Case 3: 16-year-old with significant gender dysphoria and past suicide attempts, feeling alienated with a strong focus on transitioning.

Case 4: 17-year-old facing gender dysphoria complicated by family resistance, social anxiety, and unresolved grief, destabilizing family acceptance.

Case 5: 15-year-old with major depression, social anxiety, and emergent gender dysphoria, poor medication response, and preference for solitude, indicating an uncertain prognosis.

**Conclusions:**

This study explores whether pervasive developmental disorders and gender dysphoria coexist by chance, influence each other, or share a common cause. It examines whether atypical gender identity might lie dormant and what may trigger its expression. These cases highlight the complexity of treating gender dysphoria in adolescents with developmental disorders, suggesting that tailored support and therapy can improve psychosocial outcomes and self-acceptance.

**Disclosure of Interest:**

None Declared

